# Quantification of hepatic steatosis on post-contrast computed tomography scans using artificial intelligence tools

**DOI:** 10.1007/s00261-025-05137-x

**Published:** 2025-07-26

**Authors:** Brian A. Derstine, Sven A. Holcombe, Vincent L. Chen, Manjunath P. Pai, June A. Sullivan, Stewart C. Wang, Grace L. Su

**Affiliations:** 1 Michigan Medicine, Ann Arbor, USA; 2 VA Ann Arbor Healthcare System, Ann Arbor, USA

## Abstract

**Purpose:**

Early detection of steatotic liver disease (SLD) is critically important. In clinical practice, hepatic steatosis is frequently diagnosed using computed tomography (CT) performed for unrelated clinical indications. An equation for estimating magnetic resonance proton density fat fraction (MR-PDFF) using liver attenuation on non-contrast CT exists, but no equivalent equation exists for post-contrast CT. We sought to (1) determine whether an automated workflow can accurately measure liver attenuation, (2) validate previously identified optimal thresholds for liver or liver-spleen attenuation in post-contrast studies, and (3) develop a method for estimating MR-PDFF (FF) on post-contrast CT.

**Methods:**

The fully automated TotalSegmentator ‘total’ machine learning model was used to segment 3D liver and spleen from non-contrast and post-contrast CT scans. Mean attenuation was extracted from liver (L) and spleen (S) volumes and from manually placed regions of interest (ROIs) in multi-phase CT scans of two cohorts: derivation (*n* = 1740) and external validation (*n* = 1044). Non-linear regression was used to determine the optimal coefficients for three phase-specific (arterial, venous, delayed) increasing exponential decay equations relating post-contrast L to non-contrast L. MR-PDFF was estimated from non-contrast CT and used as the reference standard.

**Results:**

The mean attenuation for manual ROIs versus automated volumes were nearly perfectly correlated for both liver and spleen (*r* > .96, *p* < .001). For moderate-to-severe steatosis (L < 40 HU), the density of the liver (L) alone was a better classifier than either liver-spleen difference (L-S) or ratio (L/S) on post-contrast CTs. Fat fraction calculated using a corrected post-contrast liver attenuation measure agreed with non-contrast FF > 15% in both the derivation and external validation cohort, with AUROC between 0.92 and 0.97 on arterial, venous, and delayed phases.

**Conclusion:**

Automated volumetric mean attenuation of liver and spleen can be used instead of manually placed ROIs for liver fat assessments. Liver attenuation alone in post-contrast phases can be used to assess the presence of moderate-to-severe hepatic steatosis. Correction equations for liver attenuation on post-contrast phase CT scans enable reasonable quantification of liver steatosis, providing potential opportunities for utilizing clinical scans to develop large scale screening or studies in SLD.

**Supplementary Information:**

The online version contains supplementary material available at 10.1007/s00261-025-05137-x.

## Introduction

Hepatic steatosis, the hallmark of steatotic liver disease (SLD) which includes both metabolic dysfunction and alcohol associated liver disease, is the most common cause of liver disease globally and a leading cause of end-stage liver disease [1–3]. Hepatic steatosis often does not present with symptoms until late in the disease with the occurrence of hepatic decompensation, at which time survival is less than 50% in 2 years [4]; thus, there is an essential need for easy screening modalities for the general population. The traditional evaluation method of liver biopsy is an invasive procedure associated with risks including bleeding, pain, and mortality and subject to sampling error due to the small amount of liver captured in the biopsy [5].

In clinical practice, steatosis is usually identified noninvasively through qualitative readings on medical imaging including ultrasonography, computed tomography (CT), and magnetic resonance imaging (MRI). Magnetic resonance imaging-derived proton density fat fraction (MR-PDFF) can most accurately quantify liver fat with high correlations compared to biopsy-proven steatosis [6], but it is expensive and not universally available [7]. MR-PDFF values can be associated with traditional biopsy definitions of > 5% (mild) and > 15% (moderate-to-severe) [8]. Alternatively, liver attenuation with or without spleen attenuation adjustment on non-contrast CT also correlates well with the degree of steatosis on biopsy [9–14]. Most studies use manually placed regions of interest (ROIs) to extract attenuation values and define steatosis based on correlation with liver biopsy [15]. Common thresholds for moderate-to-severe steatosis (MR-PDFF > 15%) included liver attenuation of < 40 HU or liver/spleen ratio of < 1.1 [13–16].

In addition to thresholds, a linear relationship between the percentage of steatosis and non-contrast liver attenuation has been demonstrated using MR-PDFF in conjunction with a phantom and an equation developed to calculate equivalent MR-PDFF from non-contrast CT liver attenuation [14]. To our knowledge, no similar equation exists for post-contrast liver attenuation. Arterial, venous, and delayed phases result in different liver and spleen attenuation values as contrast is metabolized by the body; therefore the non-contrast equation is unlikely to produce accurate estimates of MR-PDFF when used with post-contrast liver attenuation. Indeed, recently proposed post-contrast mean liver attenuation thresholds (< 90 HU venous, < 60 HU delayed) for identifying patients with moderate-to-severe hepatic steatosis are drastically different from the non-contrast threshold (< 40 HU) [17]. These post-contrast thresholds have not been validated in external datasets.

Although manual placement of ROIs has been useful for quantifying hepatic fat, the process is time-consuming and error prone. Automating the extraction of hepatic fat using artificial intelligence methodology greatly increases the speed and reproducibility of measurement. The increased speed and reproducibility from automation are required as a first step towards clinical implementation. However, the equivalence of manual ROI liver attenuation versus automated whole liver attenuation has not been established. Furthermore, while CT is more often performed than MR, especially outside large academic centers, a significant number of CTs performed for clinical indications utilize protocols that include contrast. Demonstrating the validity of automated processes for quantifying hepatic fat in post-contrast CT will greatly increase the pool of available imaging datasets.

In this study, we sought to develop an automated workflow to enable opportunistic determination of hepatic steatosis using post-contrast CT scans performed for other clinical indications. We aimed to (1) determine whether an automated workflow can accurately measure liver attenuation, (2) validate previously identified optimal thresholds for liver or liver-spleen attenuation in post-contrast studies, and (3) develop a method for estimating MR-PDFF on post-contrast CT.

## Methods

### Study population

The derivation cohort consisted of 1740 subjects drawn from two distinct sub-cohorts at a single institution: (1) a healthy reference population of adult kidney donor candidates (*n* = 1555) that had been previously studied [18], and (2) an opportunistic clinical cohort with multi-phase CT acquisitions performed in a single examination (*n* = 185). CT scans of the latter cohort were performed for issues related to hematuria (*n* = 114) and for diseases of the adrenals (*n* = 18), liver (*n* = 13), kidneys (*n* = 13), and pancreas (*n* = 2), and other reasons (*n* = 25). Patients were included if they were over 18 years of age; had CT scans performed with a GE Discovery or Lightspeed scanner, using the GE Standard convolution kernel at 120 kVp and slice thickness of 0.625 (12.9%), 1.25 (8.6%), 2.5 (29.6%), or 5 mm (48.8%), with non-contrast series plus at least one post-contrast phase (arterial, venous, delayed) imaging series in the same examination, with the full body torso visible in the axial field of view. An external validation cohort (*n* = 1044 subjects) was used to assess performance of the correction equations. This cohort had the same inclusion criteria of multi-phase CT acquisitions performed in a single examination and was drawn from a previously detailed cohort of Veterans Administration (VA) patients, which is a different institution from the derivation cohort [19]. 0.4% of CTs were performed with GE (Discovery), 63.8% Philips (Brilliance, iCT, Ingenuity, Precedence), 11.2% Siemens (Emotion, Sensation, Somatom Definition AS), 24.6% Toshiba (Acquilon), or 0.1% Picker scanners, using soft tissue convolution kernels of 80 (0.1%), 100 (5.7%), 120 (89.8%), 130 (3.0%), 135 (0.1%), or 140 (1.3%) kVp and slice thicknesses of 2 (1.7%), 3 (52.8%), 3.2 (2.1%), 5 (42.5%) mm or other values between 1 and 8 mm (0.9%).

### Image processing

Scan phase was identified as one of four categories (non-contrast, arterial, venous, or delayed) using a ResNet-50 machine learning classification model. This model was trained on 17,000 individual slices from 1,700 chest and abdomen scans of known phase designation, with scan counts balanced between sexes and between age categories of 0–8, 9–16, 17–25, then 20-year increments to 95 years. Training slices were selected to be equally spaced between vertebral levels L5 and T1 at approximately 1 slice per visible vertebra. The phase label for each scan in the current study was determined by applying this model to slices at the inferior aspect of each vertebra in each scan and then choosing the confidence-weighted majority label from this collection of single-slice classification predictions. A more detailed description of this model can be found in the Supplemental Materials.

**Fig. 1:**
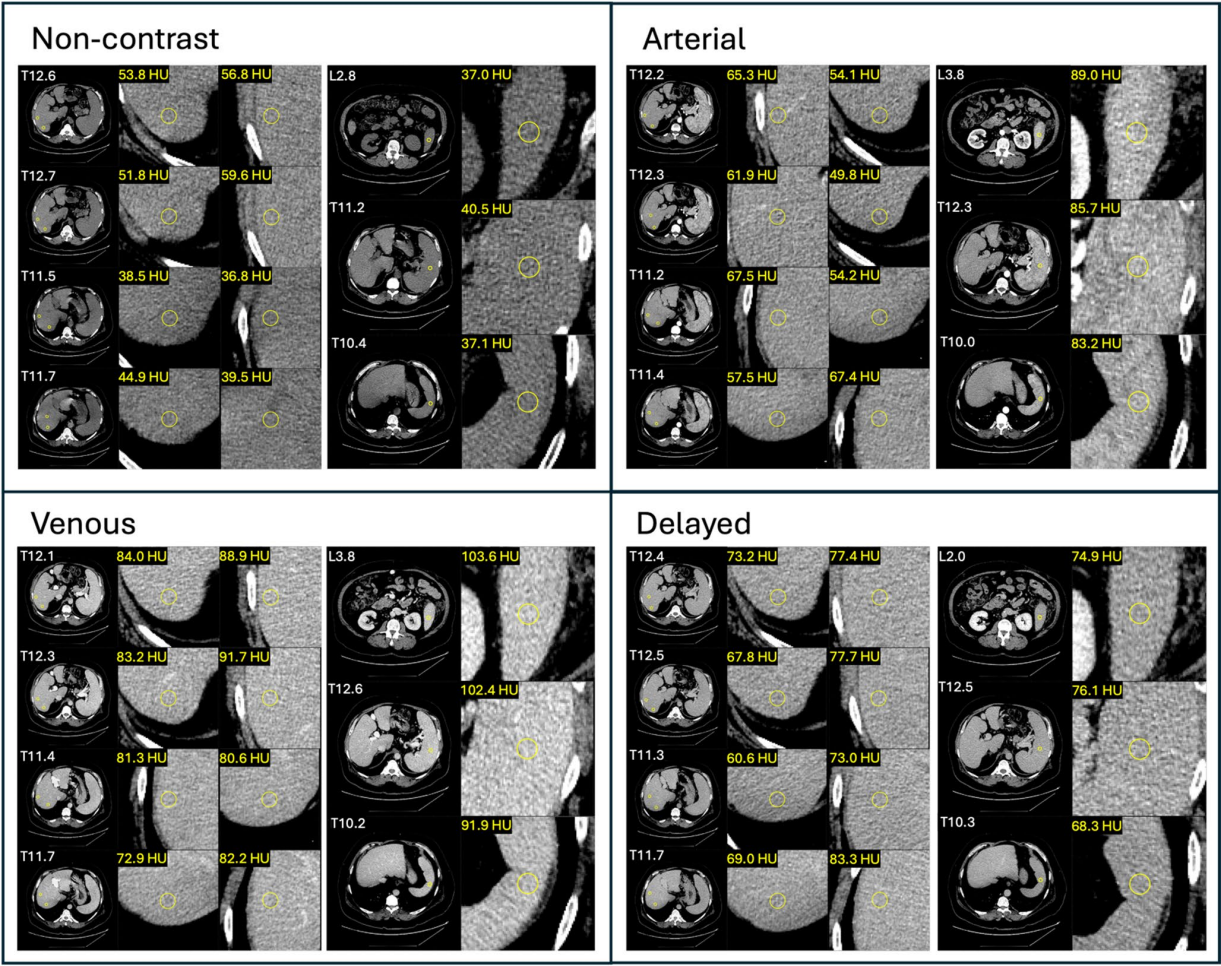
Example of manual ROI placement in liver and spleen for 67 y/o Male, BMI 34.7, Liver ROI mean attenuation (non-contrast: 47.8, arterial: 59.6, venous: 83.1, delayed: 72.8 HU), and Spleen ROI mean attenuation (non-contrast: 38.2, arterial: 86.0, venous: 99.3, delayed: 73.1 HU)

Manual 3 cm radius circular regions of interest (ROI) were placed in the liver and spleen in all CT scan phases of a randomly selected subset of all derivation cohort subjects (*n* = 527). Following prior guidance, eight ROIs were placed in the liver (two ROIs in each of liver lobes V, VI, VII, and VIII) and three ROIs in the spleen (one ROI in each of the lower, middle, and upper third) as shown in Fig. 1 [12]. Mean attenuation was calculated from all pixels across all ROIs within the liver (*L*_*roi*_) and spleen (*S*_*roi*_).

**Fig. 2:**
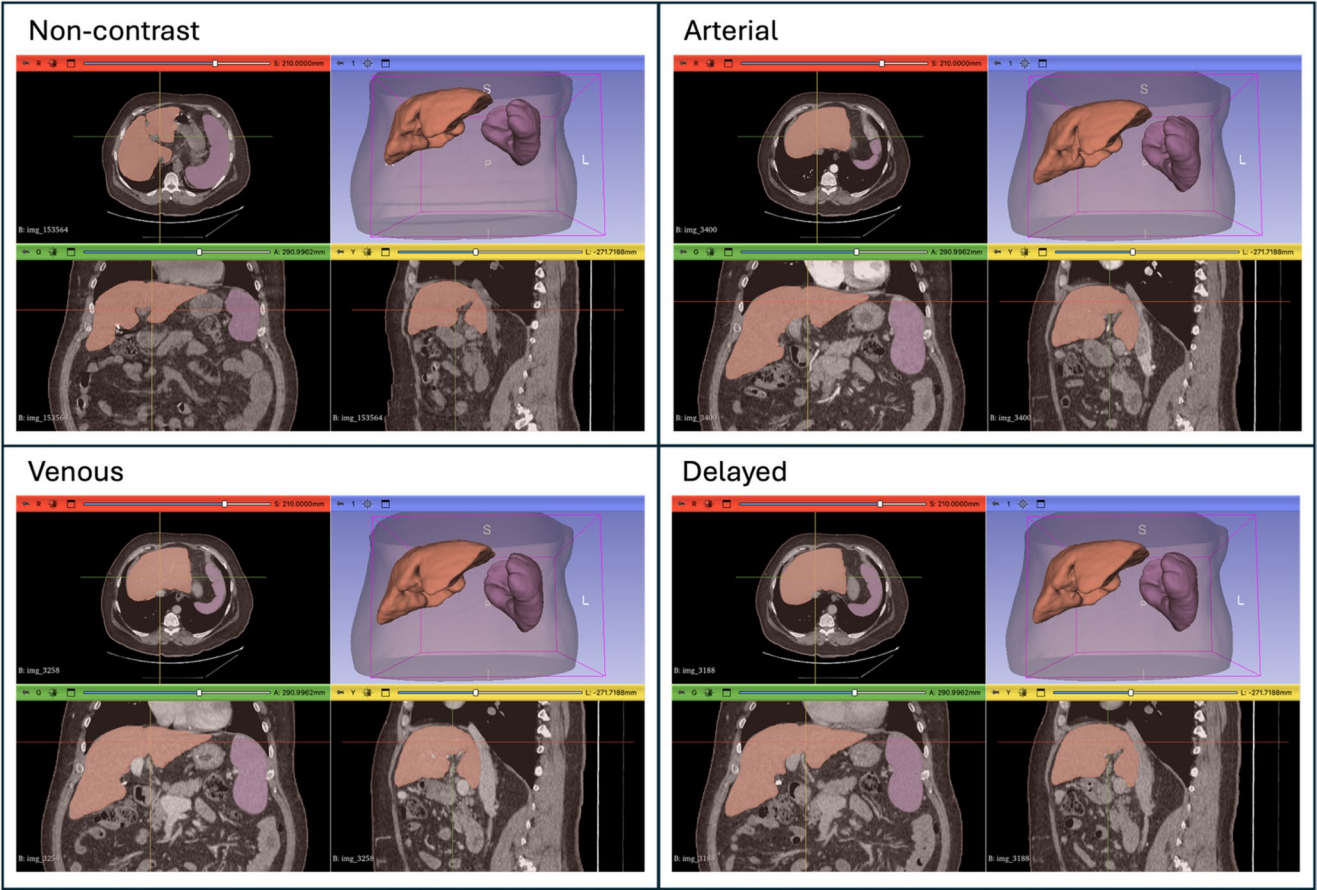
Example of automated 3D liver and spleen segmentation for 67 y/o Male, BMI 34.7, Liver 3D mean attenuation (non-contrast: 42.8, arterial: 61.2, venous: 83.1, delayed: 75.6 HU), and Spleen ROI mean attenuation (non-contrast: 34.2, arterial: 81.5, venous: 96.1, delayed: 73.0 HU)

Automated 3D volumetric segmentation of liver and spleen was performed using TotalSegmentator ‘total’ model version 2.4.0 [20]. TotalSegmentator is a publicly available convolutional neural network model that allows for robust segmentation of multiple structures on CT scans including liver and spleen, as shown in Fig. 2. Mean attenuation of automated liver (*L*_*vol*_) and spleen (*S*_*vol*_) volumes was calculated from all 3D voxels classified as ‘liver’ and ‘spleen’. Liver-spleen difference (L-S) was calculated as *L-S* = (*L*_*vol*_ *−S*_*vol*_), and liver/spleen ratio (L/S) was calculated as *L/S* = (*L*_*vol*_*/S*_*vol*_).

### Statistical methods

The Shapiro-Wilk test was used to test variable distributions for Normality and Spearman correlations and scatter plots were used to assess the linear relationship between manual and automated measures, and to compare automated measures with age, height, weight, and BMI.

Moderate-to-severe hepatic steatosis was defined for each individual based on a non-contrast liver attenuation threshold of < 40 HU, the reference standard [14]. Receiver operating characteristic (ROC) curves were calculated for L, L-S, and L/S separately for each scan phase to determine their ability to classify moderate-to-severe hepatic steatosis. Within each phase, ROC curves were compared using the DeLong method. Area under the ROC (AUROC) was calculated using the trapezoidal rule.

Using paired non-contrast and contrast scans we fit linear, logarithmic, exponential decay, smoothing splines, and second and third-degree polynomial regression models to the data. The exponential decay form was chosen because it (a) fit the data better (lower root mean squared errors, homoskedastic residuals) than the linear form, (b) was non-decreasing (whereas logarithmic, polynomial, and spline models were overfit and decreased at high values), and (c) had a parametric model form (unlike non-parametric splines), making the equation easy to describe and share.

We used the ‘nls’ function in R to determine the non-linear least-squares estimates of the three unknown coefficients for three phase-specific (arterial, venous, delayed) increasing exponential decay equations relating post-contrast mean liver attenuation ($\:{\mathrm{L}}^{{\prime\:}}$) to non-contrast L: $\:(L=\alpha\:+\beta\:*\left(1-{e}^{\left(-{L}^{{\prime\:}}*\kappa\:\right)}\right)).$ Equivalent MR-PDFF fat fraction (FF) was calculated using the equation: $\:FF=(-.58*L+38.2)$ [14] using the non-contrast L and the corrected post-contrast values of L. Agreement between non-contrast FF > 15% and corrected post-contrast FF was assessed using ROC curves in both the derivation cohort and validation cohort. Established relevant post-contrast thresholds for L, L-S, and L/S were analyzed for association with moderate hepatic steatosis, and thresholds for corrected FF were analyzed for association with FF > 15%. Sensitivity, specificity, balanced accuracy (mean of sensitivity and specificity), positive predictive value (PPV), and negative predictive value (NPV) were calculated for each threshold. Optimal thresholds were determined using balanced accuracy.

An alpha level of 0.01 was used to determine statistical significance. All statistical tests were performed in R version 4.3.2 [21], using the package ‘ggplot2’ [22] for data visualization, and the ‘pROC’ package for ROC analyses [23].

## Results

The derivation cohort was composed of 61% women with a mean (SD) age of 43.1 (12.8), weight of 80.4 (18.1) kg, height of 1.69 (0.09) m, and BMI of 27.8 (5.21) kg/m^2^. 7.6% had moderate-to-severe steatosis based on the non-contrast reference standard (L_vol_ < 40 HU).

Manually placed ROI mean attenuation was significantly correlated with automated volumetric mean attenuation values (r: 0.97 (liver), 0.97 (spleen), *p* < .001), regardless of scan phase (Table 1 and Figure S1). For CT scans that only included partial liver or spleen volumes (top and/or bottom were not visible on CT scan), the correlation remained high (r: 0.96 (liver), 0.97 (spleen), *p* < .001). As expected, liver and spleen volumetric mean attenuation demonstrated low to moderate negative correlation with age (r: −0.313 to −0.006) and height (r: −0.384 to −0.101), but moderately strong negative correlation with weight (r: −0.73 to −0.454, *p* < .001) and BMI (r: −0.693 to −0.375, *p* < .001) (Table S1 and Figure S2) in all scan phases.

The mean volumetric liver enhancement (L) for the derivation cohort was 55.7 HU (non-contrast), 68.6 HU (arterial), 90.7 HU (portal venous), and 69.6 HU (delayed). Patients with moderate-to-severe steatosis demonstrated significantly lower L compared to those with mild or no steatosis in all phases:

- Non-contrast: 28.8 (moderate-to-severe) vs. 57.9 (mild or no) HU (*p* < .001), mean difference 29.1 HU [95% CI: 27.2–31.0].
- Arterial: 40.9 vs. 69.7 HU (*p* < .001), mean difference 28.8 HU [95% CI: 25.2–32.5].
- Venous: 63.7 vs. 107.3 HU (*p* < .001), mean difference 43.5 HU [95% CI: 38.0–49.1].
- Delayed: 44.4 vs. 84.4 HU (*p* < .001), mean difference 40.0 HU [95% CI: 35.7–44.2].

**Table 1:** Derivation cohort: Spearman correlation coefficient (r) and p-value (*p*) for ROI versus 3D volumetric mean attenuation of liver and spleen showing all phases combined and split by phase

			Liver			Spleen	
Type	Phase	N	r	*p*	N	r	*p*
Complete organ volume	all	196	0.97	**< 0.001**	383	0.97	**< 0.001**
	non-contrast	101	0.89	**< 0.001**	229	0.83	**< 0.001**
	arterial	40	0.84	**< 0.001**	89	0.87	**< 0.001**
	venous	27	0.99	**< 0.001**	30	0.97	**< 0.001**
	delayed	28	0.99	**< 0.001**	35	0.97	**< 0.001**
Partial organ volume	all	639	0.96	**< 0.001**	449	0.97	**< 0.001**
	non-contrast	342	0.94	**< 0.001**	214	0.77	**< 0.001**
	arterial	190	0.93	**< 0.001**	139	0.87	**< 0.001**
	venous	18	0.98	**< 0.001**	15	0.99	**< 0.001**
	delayed	89	0.99	**< 0.001**	81	0.96	**< 0.001**

Type splits the dataset on whether the complete versus partial organ volume was visible in the scan field of view. P-values less than 0.01 shown in bold

**Fig. 3:**
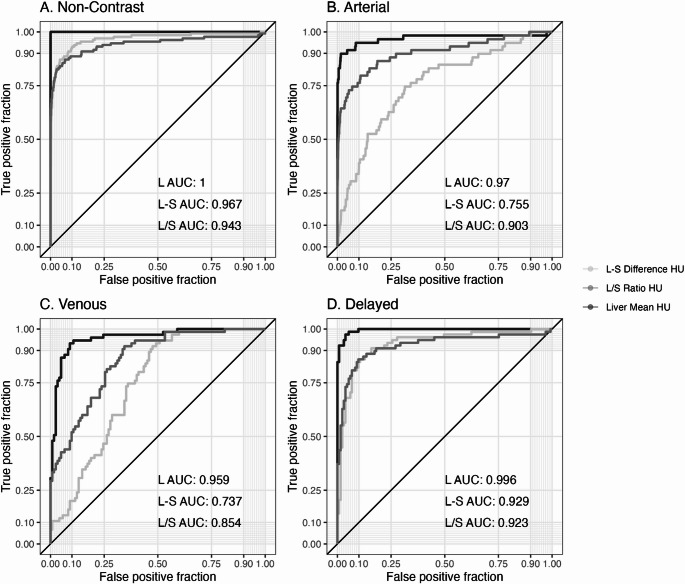
Derivation cohort: AUROC curves for classifying moderate hepatic steatosis using mean liver attenuation (L), liver-spleen attenuation difference (‘L-S’), and liver/spleen ratio (‘L/S’) in the different phase scans (non-contrast, arterial, venous, and delayed). Non-contrast L < 40 HU is the reference

Using non-contrast L < 40 HU as the reference, the AUROC for classifying moderate steatosis with L was significantly higher than L–S and L/S in venous (L: 0.959, L–S: 0.737, L/S: 0.854, L–S *p* < .001) and delayed (L: 0.996, L-S: 0.929, L/S: 0.923, *p* < .002) phases, but only significantly better than L–S on arterial (L: 0.970, L–S: 0.755, L/S: 0.903) phase (Fig. 3). Optimal L thresholds (sensitivity/specificity) were 55 HU (91.5%/95.3%) for the arterial phase, 85 HU (94.7%/89.3%) for the venous phase, and 60 HU (97.4%/96.2%) for the delayed phase (Table 2). Optimal L-S thresholds (sensitivity/specificity) were 5 HU (88.6%/92.5%) for the non-contrast phase, −40 HU (74.6%/67.6%) for the arterial phase, −15 HU (92%/50.8%) for the venous phase, and 0 HU (87.2%/86.5%) for the delayed phase (Table 3). Optimal L/S thresholds (sensitivity/specificity) were < 1.1 (86.4%/93.2%) for the non-contrast phase, < 0.6 (86.4%/78.6%) for the arterial phase, < 0.8 (86.7%/68%) for the venous phase, and < 1.0 (87.2%/86.5%) for the delayed phase (Table 4). In general, classification of moderate-to-severe hepatic steatosis was more accurate for L thresholds than for L-S or L/S thresholds.

**Table 2:** Derivation cohort: balanced accuracy (BA), sensitivity, specificity, positive predictive value (PPV), and negative predictive value (NPV) for different thresholds of mean liver attenuation (L) across post-contrast phases

Phase	L Threshold (HU)	BA	Sensitivity	Specificity	PPV	NPV
arterial	< 40	67.8	35.6 (21/59)	100 (1505/1505)	100 (21/21)	97.5 (1505/1543)
	< 45	81.4	62.7 (37/59)	100 (1505/1505)	100 (37/37)	98.6 (1505/1527)
	< 50	91.7	84.7 (50/59)	98.7 (1486/1505)	72.5 (50/69)	99.4 (1486/1495)
	**< 55**	**93.4**	**91.5 (54/59)**	**95.3 (1434/1505)**	**43.2 (54/125)**	**99.7 (1434/1439)**
	< 60	91.2	94.9 (56/59)	87.4 (1316/1505)	22.9 (56/245)	99.8 (1316/1319)
	< 65	83.8	96.6 (57/59)	70.9 (1067/1505)	11.5 (57/495)	99.8 (1067/1069)
	< 70	72.4	98.3 (58/59)	46.4 (699/1505)	6.7 (58/864)	99.9 (699/700)
venous	< 70	78.8	60 (45/75)	97.5 (119/122)	93.8 (45/48)	79.9 (119/149)
	< 75	85.4	73.3 (55/75)	97.5 (119/122)	94.8 (55/58)	85.6 (119/139)
	< 80	88.2	81.3 (61/75)	95.1 (116/122)	91 (61/67)	89.2 (116/130)
	**< 85**	**92.0**	**94.7 (71/75)**	**89.3 (109/122)**	**84.5 (71/84)**	**96.5 (109/113)**
	< 90	88.3	94.7 (71/75)	82 (100/122)	76.3 (71/93)	96.2 (100/104)
	< 95	85.6	97.3 (73/75)	73.8 (90/122)	69.5 (73/105)	97.8 (90/92)
	< 100	78.6	97.3 (73/75)	59.8 (73/122)	59.8 (73/122)	97.3 (73/75)
delayed	< 45	69.2	38.5 (30/78)	100 (133/133)	100 (30/30)	73.5 (133/181)
	< 50	78.2	56.4 (44/78)	100 (133/133)	100 (44/44)	79.6 (133/167)
	< 55	95.1	91 (71/78)	99.2 (132/133)	98.6 (71/72)	95 (132/139)
	**< 60**	**96.8**	**97.4 (76/78)**	**96.2 (128/133)**	**93.8 (76/81)**	**98.5 (128/130)**
	< 65	94.0	100 (78/78)	88 (117/133)	83 (78/94)	100 (117/117)
	< 70	86.5	100 (78/78)	72.9 (97/133)	68.4 (78/114)	100 (97/97)
	< 75	81.2	100 (78/78)	62.4 (83/133)	60.9 (78/128)	100 (83/83)

Values shown as percentages with numerator and denominator counts in parentheses. Rows with maximum balanced accuracy shown in bold for each phase. Non-contrast L < 40 HU is the reference

**Table 3:** Derivation cohort: balanced accuracy (BA), sensitivity, specificity, positive predictive value (PPV), and negative predictive value (NPV) for different thresholds of liver-spleen attenuation difference (‘L–S’) across non-contrast and post-contrast phases

Phase	‘L–S’ Threshold (HU)	BA	Sensitivity	Specificity	PPV	NPV
non-contrast	< −15	63.3	26.5 (35/132)	100 (1608/1608)	100 (35/35)	94.3 (1608/1705)
	< −10	69.3	38.6 (51/132)	99.9 (1607/1608)	98.1 (51/52)	95.2 (1607/1688)
	< −5	81.9	64.4 (85/132)	99.4 (1598/1608)	89.5 (85/95)	97.1 (1598/1645)
	< 0	88.3	78.8 (104/132)	97.8 (1572/1608)	74.3 (104/140)	98.2 (1572/1600)
	**< 5**	**90.6**	**88.6 (117/132)**	**92.5 (1488/1608)**	**49.4 (117/237)**	**99 (1488/1503)**
	< 10	86.2	97 (128/132)	75.4 (1213/1608)	24.5 (128/523)	99.7 (1213/1217)
	< 15	68.9	98.5 (130/132)	39.2 (631/1608)	11.7 (130/1107)	99.7 (631/633)
arterial	< −55	62.1	33.9 (20/59)	90.4 (1360/1505)	12.1 (20/165)	97.2 (1360/1399)
	< −50	68.7	52.5 (31/59)	84.9 (1278/1505)	12 (31/258)	97.9 (1278/1306)
	< −45	68.5	59.3 (35/59)	77.7 (1170/1505)	9.5 (35/370)	98 (1170/1194)
	**< −40**	**71.1**	**74.6 (44/59)**	**67.6 (1017/1505)**	**8.3 (44/532)**	**98.5 (1017/1032)**
	< −35	69.5	81.4 (48/59)	57.6 (867/1505)	7 (48/686)	98.7 (867/878)
	< −30	64.3	84.7 (50/59)	43.9 (660/1505)	5.6 (50/895)	98.7 (660/669)
	< −25	60.8	89.8 (53/59)	31.8 (478/1505)	4.9 (53/1080)	98.8 (478/484)
venous	< −30	63.4	54.7 (41/75)	72.1 (88/122)	54.7 (41/75)	72.1 (88/122)
	< −25	63.5	61.3 (46/75)	65.6 (80/122)	52.3 (46/88)	73.4 (80/109)
	< −20	68.8	78.7 (59/75)	59 (72/122)	54.1 (59/109)	81.8 (72/88)
	**< −15**	**71.4**	**92 (69/75)**	**50.8 (62/122)**	**53.5 (69/129)**	**91.2 (62/68)**
	< −10	70.4	97.3 (73/75)	43.4 (53/122)	51.4 (73/142)	96.4 (53/55)
	< −5	65.7	98.7 (74/75)	32.8 (40/122)	47.4 (74/156)	97.6 (40/41)
	< 0	61.6	98.7 (74/75)	24.6 (30/122)	44.6 (74/166)	96.8 (30/31)
delayed	< −15	68.5	38.5 (30/78)	98.5 (131/133)	93.8 (30/32)	73.2 (131/179)
	< −10	76.7	56.4 (44/78)	97 (129/133)	91.7 (44/48)	79.1 (129/163)
	< −5	85.1	76.9 (60/78)	93.2 (124/133)	87 (60/69)	87.3 (124/142)
	**< 0**	**86.8**	**87.2 (68/78)**	**86.5 (115/133)**	**79.1 (68/86)**	**92 (115/125)**
	< 5	83.5	94.9 (74/78)	72.2 (96/133)	66.7 (74/111)	96 (96/100)
	< 10	72.8	97.4 (76/78)	48.1 (64/133)	52.4 (76/145)	97 (64/66)
	< 15	60.6	98.7 (77/78)	22.6 (30/133)	42.8 (77/180)	96.8 (30/31)

Values shown as percentages with numerator and denominator counts in parentheses. Rows with maximum balanced accuracy shown in bold for each phase. Non-contrast L < 40 HU is the reference

**Table 4:** Derivation cohort: balanced accuracy (BA), sensitivity, specificity, positive predictive value (PPV), and negative predictive value (NPV) for different thresholds of liver/spleen attenuation ratio (‘L/S’) across non-contrast and post-contrast phases

Phase	‘L/S’ Threshold	BA	Sensitivity	Specificity	PPV	NPV
non-contrast	< 0.8	75.7	51.5 (68/132)	99.9 (1607/1608)	98.6 (68/69)	96.2 (1607/1671)
	< 0.9	84.5	69.7 (92/132)	99.3 (1597/1608)	89.3 (92/103)	97.6 (1597/1637)
	< 1	88.3	78.8 (104/132)	97.8 (1572/1608)	74.3 (104/140)	98.2 (1572/1600)
	**< 1.1**	**89.8**	**86.4 (114/132)**	**93.2 (1498/1608)**	**50.9 (114/224)**	**98.8 (1498/1516)**
	< 1.2	85.9	93.2 (123/132)	78.5 (1263/1608)	26.3 (123/468)	99.3 (1263/1272)
	< 1.3	73.0	95.5 (126/132)	50.6 (813/1608)	13.7 (126/921)	99.3 (813/819)
arterial	< 0.3	56.8	13.6 (8/59)	100 (1505/1505)	100 (8/8)	96.7 (1505/1556)
	< 0.4	63.6	27.1 (16/59)	100 (1505/1505)	100 (16/16)	97.2 (1505/1548)
	< 0.5	81.1	64.4 (38/59)	97.9 (1473/1505)	54.3 (38/70)	98.6 (1473/1494)
	**< 0.6**	**82.5**	**86.4 (51/59)**	**78.6 (1183/1505)**	**13.7 (51/373)**	**99.3 (1183/1191)**
	< 0.7	67.8	93.2 (55/59)	42.5 (639/1505)	6 (55/921)	99.4 (639/643)
	< 0.8	57.1	98.3 (58/59)	15.9 (240/1505)	4.4 (58/1323)	99.6 (240/241)
venous	< 0.5	56.7	13.3 (10/75)	100 (122/122)	100 (10/10)	65.2 (122/187)
	< 0.6	64.3	29.3 (22/75)	99.2 (121/122)	95.7 (22/23)	69.5 (121/174)
	< 0.7	71.4	56 (42/75)	86.9 (106/122)	72.4 (42/58)	76.3 (106/139)
	**< 0.8**	**77.3**	**86.7 (65/75)**	**68 (83/122)**	**62.5 (65/104)**	**89.2 (83/93)**
	< 0.9	70.6	98.7 (74/75)	42.6 (52/122)	51.4 (74/144)	98.1 (52/53)
	< 1	61.6	98.7 (74/75)	24.6 (30/122)	44.6 (74/166)	96.8 (30/31)
delayed	< 0.7	62.8	25.6 (20/78)	100 (133/133)	100 (20/20)	69.6 (133/191)
	< 0.8	72.3	46.2 (36/78)	98.5 (131/133)	94.7 (36/38)	75.7 (131/173)
	< 0.9	85.8	76.9 (60/78)	94.7 (126/133)	89.6 (60/67)	87.5 (126/144)
	**< 1**	**86.8**	**87.2 (68/78)**	**86.5 (115/133)**	**79.1 (68/86)**	**92 (115/125)**
	< 1.1	77.5	94.9 (74/78)	60.2 (80/133)	58.3 (74/127)	95.2 (80/84)
	< 1.2	61.6	96.2 (75/78)	27.1 (36/133)	43.6 (75/172)	92.3 (36/39)

Values shown as percentages with numerator and denominator counts in parentheses. Rows with maximum balanced accuracy shown in bold for each phase. Non-contrast L < 40 HU is the reference

**Fig. 4:**
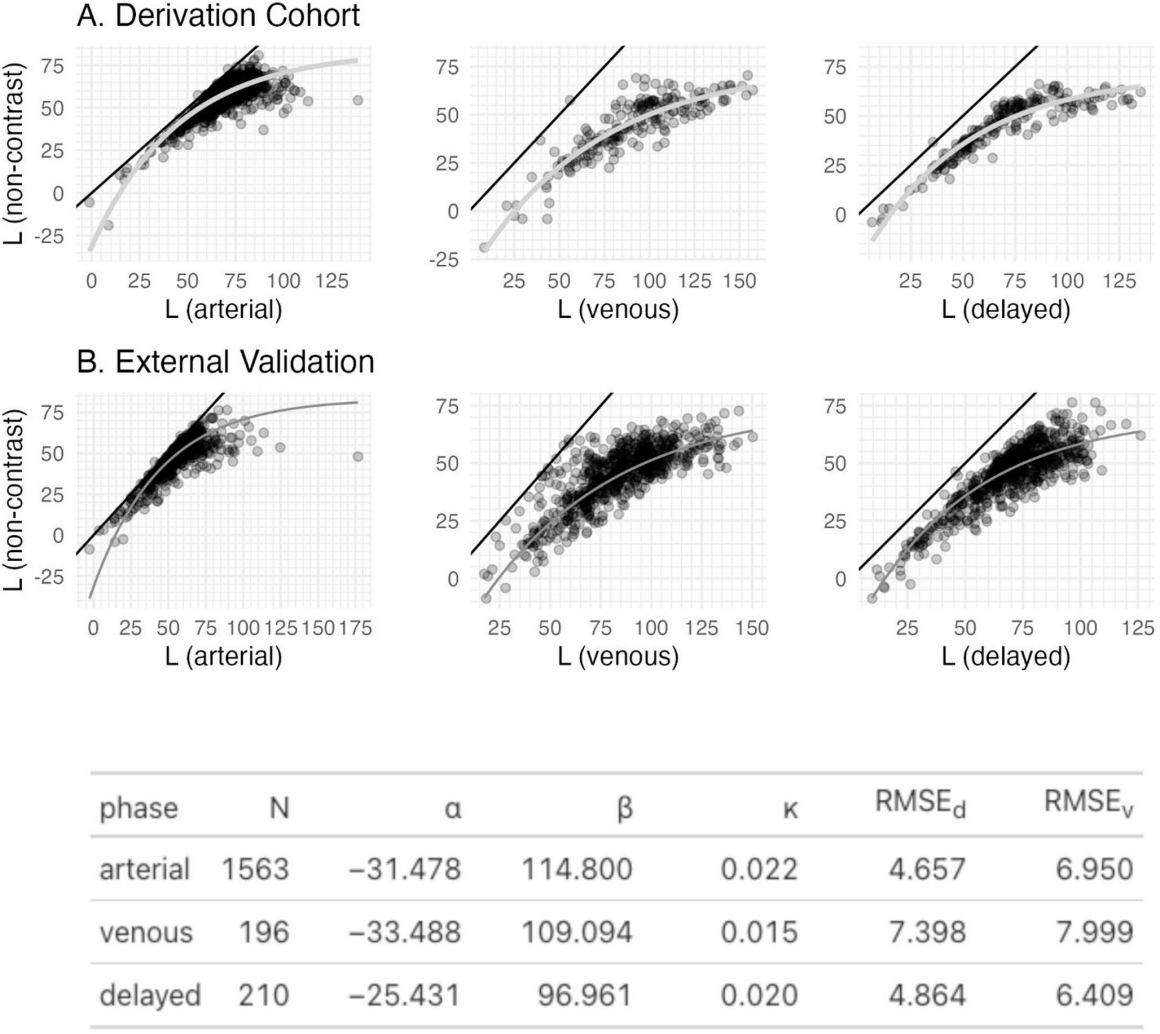
Scatter plots showing paired scans with post-contrast L value on the x-axis and non-contrast L value on the y-axis, line of equivalence (black), best-fit non-linear (increasing exponential decay) regression equation (light grey, derivation cohort), and the same regression equation overlaid on the external validation dataset (dark grey): $\:Corrected\:L=\alpha\:+\beta\:*(1-{e}^{(-L*\kappa\:)})$, where L is the post-contrast volumetric mean liver attenuation. Table lists regression equation coefficients and root mean squared error (RMSE) for the derivation (d) and validation (v) cohorts by phase

Correction equations for converting L from each contrast phase into a non-contrast equivalent value are as follows (Fig. 4A):

- arterial: Corrected L = −31.478 + 114.8 × (1−e^(−L × 0.022)^)
- venous: Corrected L = −33.488 + 109.094 × (1−e^(−L × 0.015)^)
- delayed: Corrected L = −25.431 + 96.961 × (1−e^(−L × 0.02)^)

The root mean squared errors (RMSE) for these equations were comparable between the derivation vs. external validation sets (arterial: 4.7 vs. 6.9, venous: 7.4 vs. 8.0, delayed: 4.9 vs. 6.4) and the regression equation predictions overlaid on the external validation set demonstrated strong visual concordance between derivation and validation sets overall (Fig. 4) and when split by manufacturer (Figure S3).

**Fig. 5:**
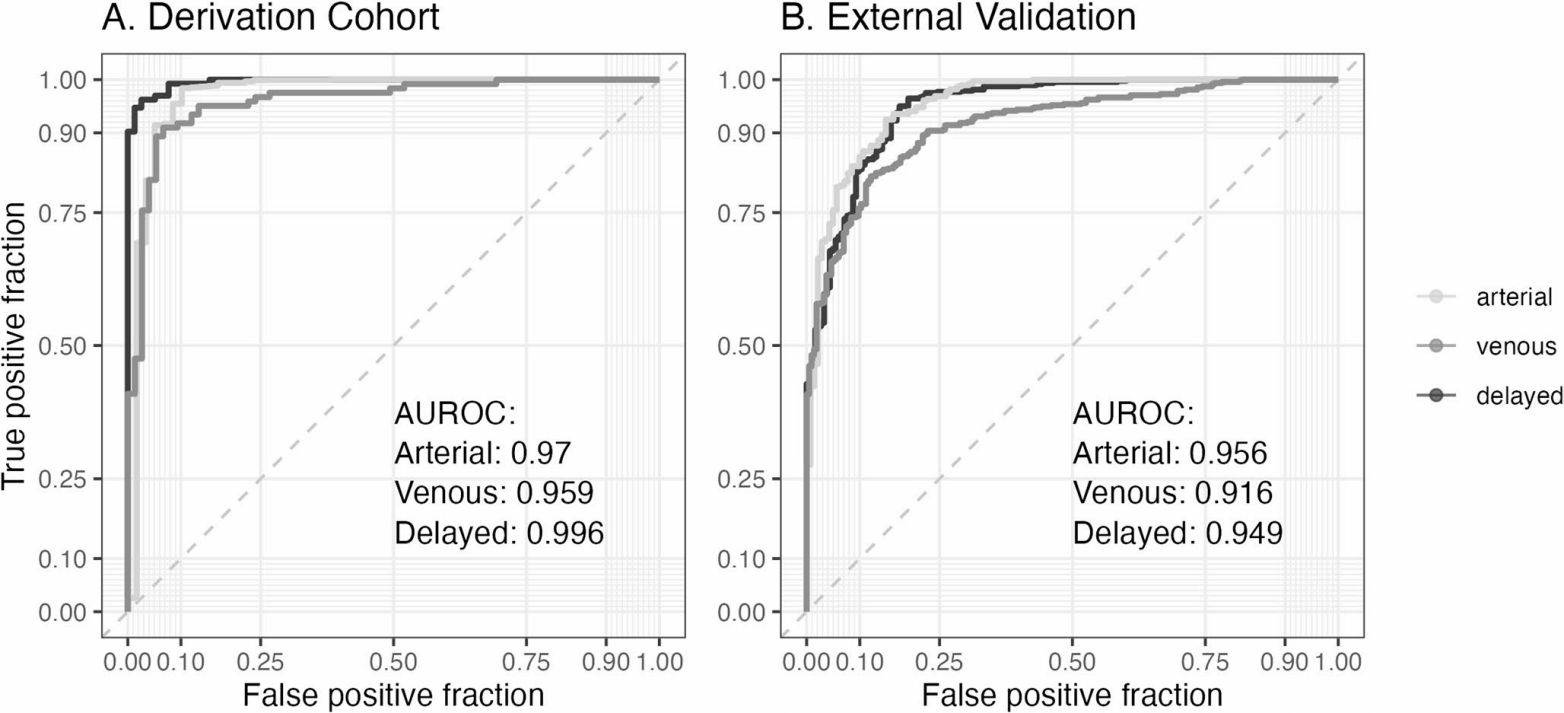
AUROC curves for classifying moderate hepatic steatosis using FF calculated from corrected post-contrast L values on arterial, venous, and delayed phase. Non-contrast FF > 15% is the reference

When using FF > 15% on non-contrast CT as the reference, the AUROC for classifying moderate steatosis using FF from corrected post-contrast liver attenuation was 0.97, 0.959, and 0.996 in the derivation cohort (Fig. 5A), and 0.96, 0.92, 0.95 in the external validation cohort (Fig. 5B) for arterial, venous, and delayed phase scans respectively. The > 15% threshold was optimal for post-correction venous and delayed phase scans, while > 10% was optimal for arterial scans (Table 5).

**Table 5:** Derivation cohort: balanced accuracy (BA), sensitivity, specificity, positive predictive value (PPV), and negative predictive value (NPV) for different thresholds of liver fat fraction (FF) across non-contrast (reference) and post-contrast phases

Phase	FF Threshold (%)	BA	Sensitivity	Specificity	PPV	NPV
non-contrast	> 5	78.9	57.7 (928/1608)	100 (132/132)	100 (928/928)	16.3 (132/812)
	> 10	94.9	89.8 (1444/1608)	100 (132/132)	100 (1444/1444)	44.6 (132/296)
	**> 15**	**100.0**	**100 (1608/1608)**	**100 (132/132)**	**100 (1608/1608)**	**100 (132/132)**
	> 20	74.2	100 (1608/1608)	48.5 (64/132)	95.9 (1608/1676)	100 (64/64)
arterial	> 5	78.7	59.1 (890/1505)	98.3 (58/59)	99.9 (890/891)	8.6 (58/673)
	**> 10**	**92.7**	**95.6 (1439/1505)**	**89.8 (53/59)**	**99.6 (1439/1445)**	**44.5 (53/119)**
	> 15	78.0	100 (1505/1505)	55.9 (33/59)	98.3 (1505/1531)	100 (33/33)
	> 20	63.6	100 (1505/1505)	27.1 (16/59)	97.2 (1505/1548)	100 (16/16)
venous	> 5	61.1	22.1 (27/122)	100 (75/75)	100 (27/27)	44.1 (75/170)
	> 10	83.1	68.9 (84/122)	97.3 (73/75)	97.7 (84/86)	65.8 (73/111)
	**> 15**	**85.7**	**96.7 (118/122)**	**74.7 (56/75)**	**86.1 (118/137)**	**93.3 (56/60)**
	> 20	70.3	99.2 (121/122)	41.3 (31/75)	73.3 (121/165)	96.9 (31/32)
delayed	> 5	62.4	24.8 (33/133)	100 (78/78)	100 (33/33)	43.8 (78/178)
	> 10	84.6	69.2 (92/133)	100 (78/78)	100 (92/92)	65.5 (78/119)
	**> 15**	**95.4**	**98.5 (131/133)**	**92.3 (72/78)**	**95.6 (131/137)**	**97.3 (72/74)**
	> 20	69.2	100 (133/133)	38.5 (30/78)	73.5 (133/181)	100 (30/30)

Values shown as percentages with numerator and denominator counts in parentheses. Rows with maximum balanced accuracy shown in bold for each phase. Non-contrast FF > 15% is the reference

To assess the potential impact of using FF, we examined the radiologist reports on 1715 non-contrast CT scans in the derivation cohort with available reports for review. Of these, 10.6% had a report of hepatic steatosis based on a previously validated natural language processing algorithm [24], while 7.6%, 17.1%, 46.1% had FF > 15%, 10%, and 5% respectively based on estimated MR-PDFF from non-contrast CT. Using estimated MR-PDFF on non-contrast CT as the reference, the sensitivity/specificity for radiologist reported steatosis was 70.2%/94.3%, 42.3%/95.9% and 20.5%/98.1% for FF > 15%, >10%, and > 5%, showing high specificity but low sensitivity, particularly for mild steatosis. Within this cohort, 29.8%, 57.8%, and 79.4% of patients with FF > 15%, >10%, and > 5% did not have steatosis reported on the radiology report, suggesting that radiology reporting of hepatic steatosis may be most prominent when there is evidence of moderate-to-severe steatosis (> 15% FF).

## Discussion

We found near-perfect correlation between liver and spleen mean attenuation measured by artificial intelligence derived automated segmentation of whole organs and manually selected ROIs, suggesting that rapid, automated segmentation could replace manual methods without sacrificing accuracy. The optimal thresholds for the detection of moderate-to-severe hepatic steatosis in post-contrast CT were 55, 85, and 60 HU for the arterial, venous, and delayed phases, respectively, comparable to previously published thresholds for venous (90 HU) and delayed (60 HU) phases reported by Pickhardt et al. [17, 25]. No published threshold for the arterial phase was available for comparison. We developed novel equations to “convert” post-contrast liver attenuation values to non-contrast equivalent values. Given the close correlation between manual and automated methods of assessing attenuation, these equations can be used with either technique and thus have value for clinical use by those without an automated methodology.

We found that post-contrast liver attenuation alone was more predictive of moderate-to-severe steatosis than liver-spleen attenuation difference or liver/spleen attenuation ratio, consistent with what has been previously reported [25]. This suggests that liver attenuation measurement alone may be sufficient for hepatic steatosis assessment. In this study, we developed and utilized a machine learning algorithm to classify CTs into non-contrast, arterial, venous, and delayed phases, similar to work performed elsewhere [26, 27], which is of particular importance when utilizing an automated methodology with large cohort sizes. Alternatively, in clinical practice, scan phase can be easily identified manually.

In addition to providing thresholds for different post-contrast phases, we also provide conversion equations for post-contrast CT liver attenuation that enable estimation of liver fat fraction using the previously reported MR-PDFF equation developed for non-contrast CT liver attenuation [14]. With an AUROC of 0.996 for predicting moderate-to-severe hepatic steatosis the performance for the delayed phase was best, likely due to liver attenuation values returning towards their non-contrast baseline as the body has had the most time to metabolize the injected contrast in the delayed phase images. Performance was also reasonable for the arterial (AUROC 0.970) and venous (AUROC 0.959) phases suggesting the potential to use post-contrast phase CT scans for opportunistic liver fat estimation in sequential scans performed for clinical indications. This approach may offer a potential alternative to MR-PDFF for durability of response assessments of emerging therapies like resmetirom to treat metabolic dysfunction-associated fatty liver disease [28]. Sequential and repeated CT scans for cancer indications are common and often include administration of contrast. Using our novel liver attenuation correction equations enables estimates of MR-PDFF on these post-contrast phase CTs, and adds incremental value for steatosis detection in clinical care when the alternative is simple binary classification of moderate-to-severe steatosis using liver attenuation threshold values. From an epidemiological standpoint, there is significant potential to assess liver fat fraction in large populations using this methodology given that the majority of CT scans stored in hospital radiology imaging databases contain contrast. Furthermore, our use of a publicly available machine learning model for image segmentation (TotalSegmentator) demonstrates the potential for broad access to automated quantification of hepatic steatosis. With the rising prevalence of steatotic liver disease, both alcohol and metabolic dysfunction, this could represent a reasonable first step towards wider screening for SLD in patients obtaining CT scans for other indications [29].

Our study had some important limitations. Primarily, the reference standard was non-contrast CT rather than liver biopsy. However, in this era of noninvasive methodology, using retrospectively paired liver biopsy with CT would have a potential bias, and the risk of biopsy would preclude any prospective study [30]. These results were not evaluated in individuals under age 18. The correction equations developed here performed extremely well in our own external validation cohort, which consisted of a broader range of CT protocols (machines, convolution kernels, kVp, slice thickness, etc.) and a different cohort of patients than the derivation cohort. However, the validation set exhibited a high degree of concentration among certain parameters (87% at 120 kVp, and 92% at either 3- or 5-mm slice thickness). These equations should be validated in large datasets that use the less common parameters (e.g., non-120 kVp), and different scan protocols and cohorts, to confirm broader applicability. In addition, a direct analysis of post-contrast CT liver attenuation and MRI PDFF using a paired dataset should be performed for validation purposes. Furthermore, the reliability of the TotalSegmentator model used here should be investigated more. Unlike manual human segmentation, the TotalSegmentator liver and spleen segmentations we used are highly reproducible; given the same input images, the model outputs the exact same 3D segmentation very quickly. However, the model’s liver and spleen segmentations are not guaranteed to be accurate on all CT images of all patients. To better understand its reliability, the model’s liver and spleen segmentation accuracy should be evaluated in a wide range of images for patients with different disease processes and using different CT scanner settings. Finally, the distribution of fat in the liver may be highly heterogenous and, by definition, volumetric measurements of liver fat using mean pixel density will produce an averaged estimate of liver fat across the whole liver. It is not known whether and how liver fat heterogeneity matters clinically and this question deserves future investigation.

In providing an automated methodology for estimating fat fraction in both post-contrast and non-contrast CT scans, we present a method to add incremental clinical value to a scan performed for other indications. Our methodology provides an objective quantitative assessment which consistently detects SLD and could allow for earlier detection of disease for those who have already received a CT scan.

## Electronic supplementary material

Below is the link to the electronic supplementary material.

Supplementary Material 1

## Data Availability

The analytic dataset analyzed during the current study and the CT phase prediction model file have been deposited in a public repository: 10.7302/xkpk-6w96.
