# Supplementary material for: Quantification of hepatic steatosis on post-contrast computed tomography scans using artificial intelligence tools
Source: Abdom Radiol (NY). 2025 Jul 26;51(2):991–1003. doi: 10.1007/s00261-025-05137-x (PMC12929335; doi:10.1007/s00261-025-05137-x)
Supplement: Supplementary file 1 — Supplementary Material 1 [file 261_2025_5137_MOESM1_ESM.docx]

“**Quantification of hepatic steatosis on post-contrast computed tomography scans using artificial intelligence tools.”**

**Supplemental Materials**


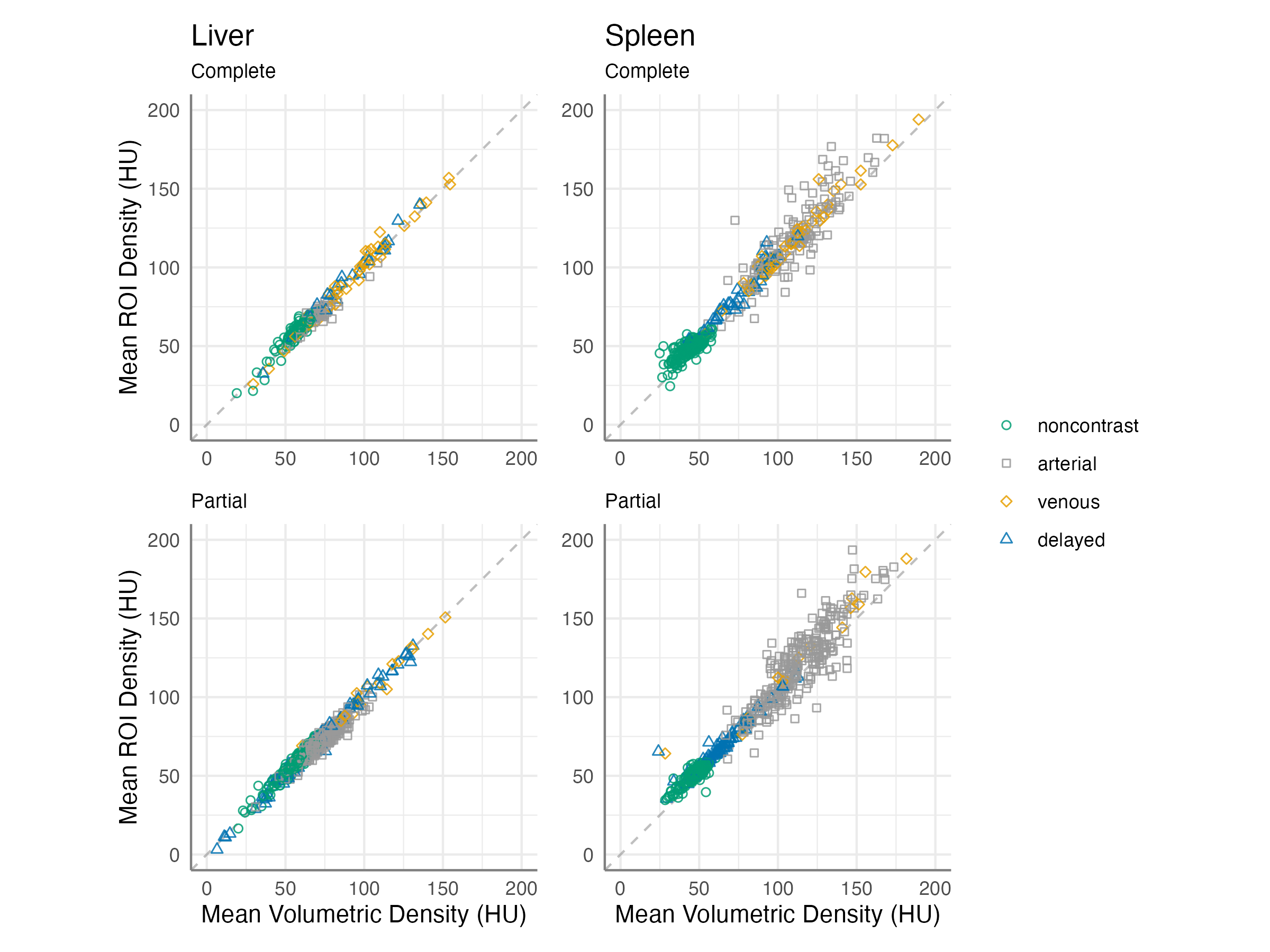


**Figure S1.** Derivation Cohort: Scatter plots of automated volumetric versus manual ROI mean attenuation with line of equivalence (dashed line), shown in different color and shape by phase.


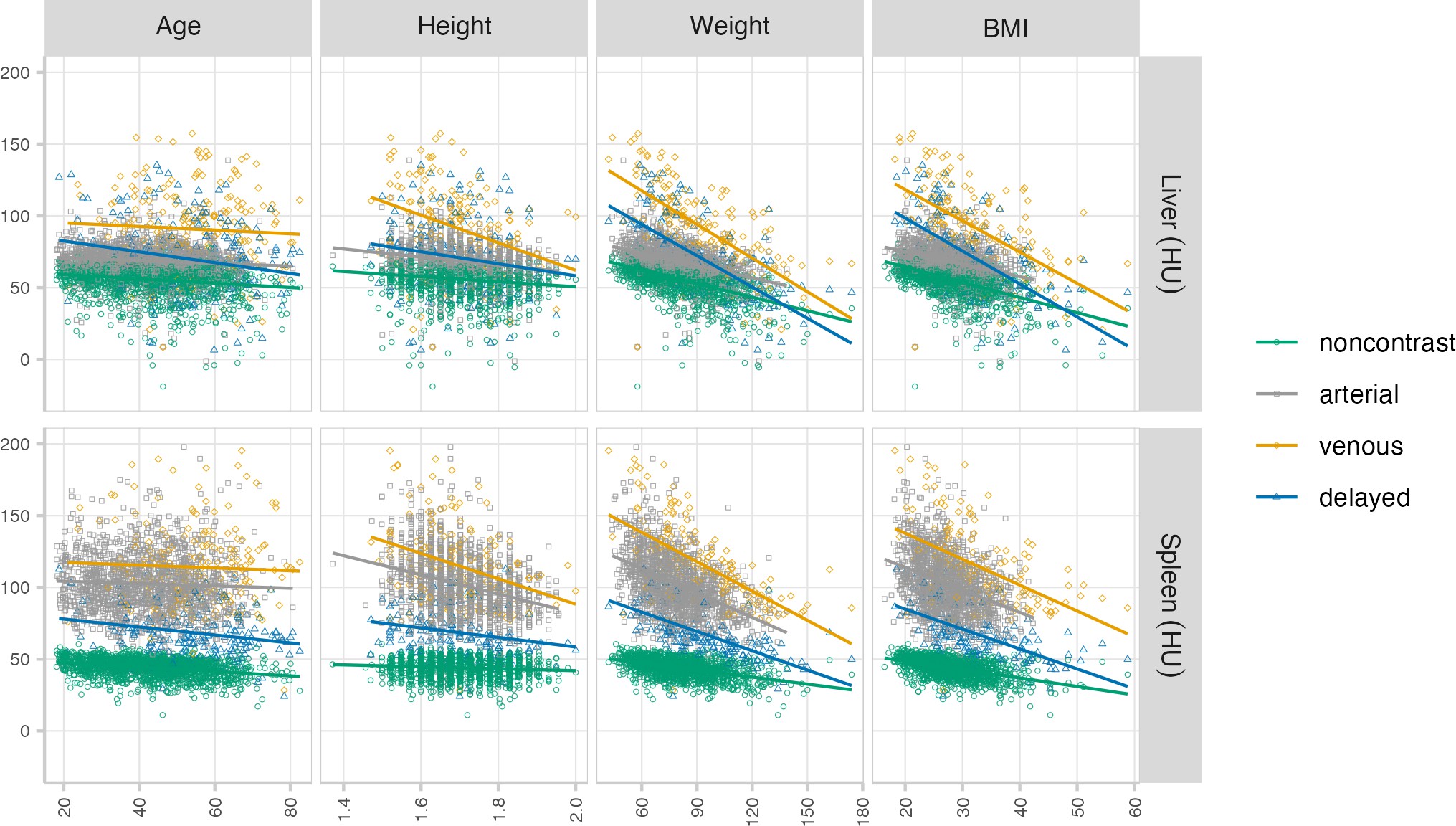


**Figure S2.** Derivation Cohort: Scatter plots of automated volumetric mean attenuation versus age (yr), height (m), weight (kg), and BMI (*kg/m*^2^) with best fit linear regression lines, shown in different color and shape by phase.


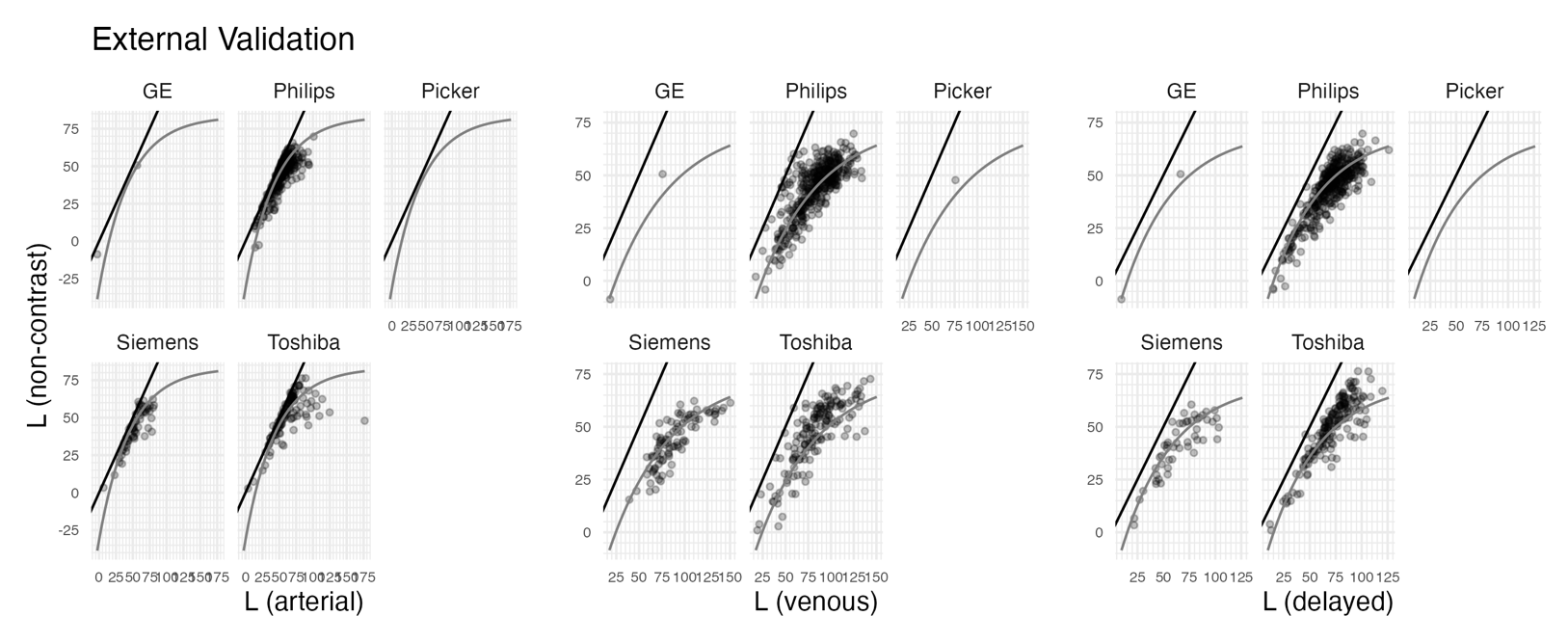


**Figure S3.** External Validation Cohort: Scatter plots showing paired scans split by CT machine manufacturer with post-contrast L value on the x-axis and non-contrast L value on the y-axis, line of equivalence (black), non-linear (increasing exponential decay) regression equation overlaid on the external validation dataset (dark grey): $\mathrm{Corrected} L=\alpha+\beta*(1-e^{(-L*\kappa)})$, where L is the post-contrast volumetric mean liver attenuation.

|  | | | Age |  |  |  | Height |  |  |  | Weight |  |  |  | BMI |  |  |
| --- | --- | --- | --- | --- | --- | --- | --- | --- | --- | --- | --- | --- | --- | --- | --- | --- | --- |
| Organ | Phase | N | r | *p* |  | N | r | *p* |  | N | r | *p* |  | N | r | *p* |  |
| Liver | non-contrast | 1740 | -0.139 | **<.001** |  | 1704 | -0.204 | **<.001** |  | 1711 | -0.546 | **<.001** |  | 1695 | -0.527 | **<.001** |  |
|  | arterial | 1564 | -0.113 | **<.001** |  | 1553 | -0.273 | **<.001** |  | 1551 | -0.467 | **<.001** |  | 1550 | -0.398 | **<.001** |  |
|  | venous | 197 | -0.047 | 0.513 |  | 164 | -0.384 | **<.001** |  | 171 | -0.711 | **<.001** |  | 157 | -0.610 | **<.001** |  |
|  | delayed | 211 | -0.126 | 0.068 |  | 177 | -0.220 | **0.003** |  | 184 | -0.732 | **<.001** |  | 168 | -0.693 | **<.001** |  |
| Spleen | non-contrast | 1740 | -0.313 | **<.001** |  | 1704 | -0.101 | **<.001** |  | 1711 | -0.454 | **<.001** |  | 1695 | -0.486 | **<.001** |  |
|  | arterial | 1564 | -0.058 | 0.021 |  | 1553 | -0.337 | **<.001** |  | 1551 | -0.482 | **<.001** |  | 1550 | -0.375 | **<.001** |  |
|  | venous | 197 | -0.006 | 0.932 |  | 164 | -0.339 | **<.001** |  | 171 | -0.635 | **<.001** |  | 157 | -0.524 | **<.001** |  |
|  | delayed | 211 | -0.165 | 0.017 |  | 177 | -0.287 | **<.001** |  | 184 | -0.728 | **<.001** |  | 168 | -0.657 | **<.001** |  |

**Table S1.** Derivation Cohort: Spearman correlation coefficients (r) and p-values (p) of automated volumetric mean attenuation versus age (yr), height (m), weight (kg), and BMI (kg/m2) split by phase. P-values less than .01 shown in bold.

**Description of Scan Phase classification model training and validation.**

CT scan phases are often broadly categorized as either non-contrast or post-contrast, however, within the post-contrast phase exist multiple different sub-categories corresponding to different image acquisition times following intravenous contrast injection as has been described in the literature [1][2]. For the purposes of the current machine learning model, we classified phase into four categories: non-contrast, arterial, venous, or delayed phase.

A dataset of 1,693 CT scans performed between 1999 and 2022 at a single institution having scan counts balanced between sexes and between age categories of 0-8, 9-16, 17-25, then 20-year increments to 95 years was identified. Scans included a broad range of manufacturers (GE, Philips, Siemens, and Toshiba), kVp (80-140), and slice thickness (0.5 – 5mm). For model training, 17,252 individual axial slices were selected from all available image slices such that for each scan, training slices would be equally spaced between vertebral levels L5 and T1 at approximately 1 slice per visible vertebra. The dataset was split into training (n=971 scans, n=9907 slices, 57.4%) and test (n=722 scans, n=7355 slices, 42.6%) sets by scan. A pre-trained ResNet-50 convolutional neural network that is 50 layers deep (‘resnet50’ function in Matlab) was used[3], with the learnable layer converted from a segmentation to a classification model with WeightLearnRateFactor and BiasLearnRateFactor both set to 10, and trained using the following options: “sgdm” solver, MiniBatchSize of 16, MaxEpochs of 10, InitialLearnRate of 3e-4, and shuffle “every-epoch”. Model inference was applied to each single-slice image to obtain class probabilities, and the class label with the maximum confidence score averaged over all slices was used as the predicted phase label for each scan (Figure S4).


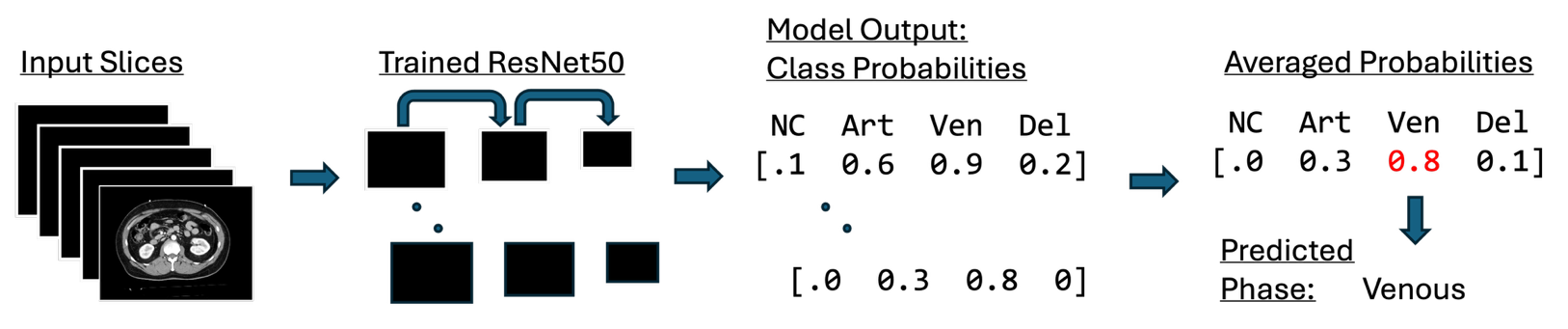


**Figure S4.** CT phase prediction model training and prediction flow. Single-slice images are input into trained ResNet50 convolutional neural network which outputs predicted probability for each of four phases: NC (non-contrast), Art (arterial), Ven (venous), Del (delayed). Class probabilities are averaged across all slices for each scan and the class with the maximum averaged probability is assigned as the phase label for that scan.

We calculated a confusion matrix for the training and testing sets to assess the number and types of classification errors by phase (Table S2). Classification accuracy was also quantified using AUROC (the “multiclass.roc” function in R). Overall accuracy was 89% in both the training and testing sets while AUROC was 0.86 for the training set and 0.87 for the testing set. By phase, non-contrast had the highest performance with a balanced accuracy (average of sensitivity and specificity) of 97%, followed by arterial (94.2%), venous (90.7%) and delayed (84.9%) in the testing set.

| Training Set | Truth: | | | |
| --- | --- | --- | --- | --- |
| Prediction: | **Arterial** | **Delayed** | **Non-contrast** | **Venous** |
| Arterial | 224 | 1 | 0 | 3 |
| Delayed | 0 | 132 | 2 | 10 |
| Non-contrast | 0 | 3 | 228 | 0 |
| Venous | 45 | 41 | 2 | 280 |
| *Sensitivity:* | *83.3%* | *74.6%* | *98.3%* | *95.6%* |
| *Specificity:* | *99.4%* | *98.5%* | *99.6%* | *87.0%* |
| *Pos Pred Value:* | *98.3%* | *91.7%* | *98.7%* | *76.1%* |
| *Neg Pred Value:* | *93.9%* | *94.6%* | *99.5%* | *97.8%* |
| *Accuracy:* | *88.9% (95% CI: 86.8-90.9%)* | | | |
|  |  | | | |
| Testing Set | Truth: | | | |
| Prediction: | **Arterial** | **Delayed** | **Non-contrast** | **Venous** |
| Arterial | 166 | 0 | 0 | 2 |
| Delayed | 0 | 98 | 7 | 15 |
| Non-contrast | 0 | 1 | 134 | 0 |
| Venous | 21 | 34 | 1 | 243 |
| *Sensitivity:* | 88.8% | 73.7% | 94.4% | 93.5% |
| *Specificity:* | 99.6% | 96.3% | 99.8% | 87.9% |
| *Pos Pred Value:* | *98.8%* | *81.7%* | *99.3%* | *81.3%* |
| *Neg Pred Value:* | *96.2%* | *94.2%* | *98.6%* | *96.0%* |
| *Accuracy:* | *88.8% (95% CI: 86.3-91.0%)* | | | |

**Table S2.** CT phase prediction model training and testing confusion matrices with one-versus-all Sensitivity and Specificity calculated by class and Overall Accuracy computed for each set.

**Supplemental References:**

[1] B. T. Dao, T. V. Nguyen, H. H. Pham, and H. Q. Nguyen, “Phase recognition in contrast‐enhanced CT scans based on deep learning and random sampling,” *Med. Phys.*, vol. 49, no. 7, pp. 4518–4528, Jul. 2022, doi: 10.1002/mp.15551.

[2] A. Anand, J. Liu, T. C. Shen, W. M. Linehan, P. A. Pinto, and R. M. Summers, “Automated classification of intravenous contrast enhancement phase of CT scans using residual networks,” in *Medical Imaging 2023: Computer-Aided Diagnosis*, K. M. Iftekharuddin and W. Chen, Eds., San Diego, United States: SPIE, Apr. 2023, p. 22. doi: 10.1117/12.2655263.

[3] He, Kaiming, Xiangyu Zhang, Shaoqing Ren, and Jian Sun. “Deep Residual Learning for Image Recognition.” In 2016 IEEE Conference on Computer Vision and Pattern Recognition (CVPR), 770–78. Las Vegas, NV, USA: IEEE, 2016, doi: 10.1109/CVPR.2016.90.
